# Process evaluation of a classroom active break (ACTI-BREAK) program for improving academic-related and physical activity outcomes for students in years 3 and 4

**DOI:** 10.1186/s12889-019-6982-z

**Published:** 2019-05-24

**Authors:** Amanda Watson, Anna Timperio, Helen Brown, Kylie D. Hesketh

**Affiliations:** Institute for Physical Activity and Nutrition (IPAN), School of Exercise and Nutrition Science, Deakin University, Geelong, Australia

**Keywords:** Intervention, Process evaluation, Children, Physical activity, Primary school

## Abstract

**Background:**

Active breaks in the classroom have been shown to be effective for increasing children’s physical activity, while simultaneously improving classroom behaviour outcomes. However, there is limited evidence on the feasibility and fidelity of these programs outside of the research context. The purpose of this study was to conduct a process evaluation to explore factors associated with feasibility and fidelity of a classroom active break (ACTI-BREAK) program designed to improve classroom behaviour and physical activity outcomes for children in primary (elementary) school Years 3 and 4.

**Methods:**

Six schools (3 intervention; 3 control) and 374 children (74% response) were included in the ACTI-BREAK pilot cluster randomised controlled trial. The intervention involved teachers implementing 3 × 5-minute moderate-intensity ACTI-BREAKS into their classroom routines, daily. This study focuses on the responses of students (*n* = 138) and their teachers (*n* = 7) who participated in the ACTI-BREAK intervention group. Intervention fidelity was assessed by number of ACTI-BREAKS completed per class per day; minutes spent in moderate-intensity physical activity (accelerometry) per ACTI-BREAK; change in physical activity from baseline to mid- and end- intervention. Intervention feasibility was explored through telephone interviews (teachers), questionnaires and focus groups (students), and teacher observations of acute effects on classroom behaviour. Qualitative data were analysed using thematic analyses; acute effects on classroom behaviour and change in physical activity were explored using paired *t*-tests; questionnaire data were described as frequencies.

**Results:**

Teachers implemented two ACTI-BREAKS/day on average, mostly of light-intensity physical activity. Physical activity increased from baseline to mid-, but not baseline to end-intervention; classroom behaviour improved immediately following ACTI-BREAKS. Barriers to implementation included ability for students to return to task and scheduling. Facilitators included ease of implementation, flexible delivery options and student enjoyment. Students were largely satisfied with the program and enjoyed ACTI-BREAKS that incorporated choice, imagination and challenge but did not enjoy ACTI-BREAKS that evoked silliness or were perceived as too difficult and some did not like doing ACTI-BREAKS in the confined space of their classroom.

**Conclusions:**

Results indicated the ACTI-BREAK program was acceptable for students and feasible for teachers, however, some minor modifications in terms of required frequency and intensity could improve fidelity.

**Trial registration:**

Australia New Zealand Clinical Trials Registry (ACTRN12617000602325). Retrospectively registered on 27 April 2017.

## Background

Physical activity during childhood is associated with multiple short and long term health benefits [1]. However, population based studies indicate less than 50% of primary (elementary) school-aged children attain the recommended 60-min of moderate- to vigorous- intensity physical activity per day required to accrue health benefits [2]. Schools are regarded as an ideal setting for the promotion of physical activity to children as children spend the majority of their waking hours at school [3]. However, physical activity interventions targeting the school environment can be difficult to implement, often due to competing time demands associated with academic accountability [4]. Active breaks are short bursts of physical activity performed in the classroom as a break from learning tasks [5]. Meta-analyses and systematic reviews show children’s classroom behaviour improves following participation in such sessions [6–8]. For example, active breaks as short as 4-min have been shown to have a positive effect on classroom behaviour immediately following participation [5, 9, 10]. Thus, active breaks may provide an attractive strategy for teachers to incrementally increase children’s daily physical activity during school hours [9, 11], while simultaneously improving classroom behaviour outcomes. While these outcome evaluation studies provide valuable insight into the effectiveness of such programs, process evaluation studies are important for exploring factors associated with intervention fidelity and feasibility [12].

Studies have explored general perceptions of active break strategies, reporting that active breaks that were short (e.g. < 5-min) and quick and easy to implement would be more likely to be adopted in daily practice [4, 13–16]. Only one previous active break intervention has performed a process evaluation to accompany the outcome evaluation [17]. That study indicated that although 10- and 20-min active breaks were effective for improving classroom behaviour, while 5-min active breaks were not, teachers considered active breaks longer than 5-min to not be feasible within a crowded curriculum [17]. This finding highlights the importance of process evaluation to provide insights into whether interventions would be feasible and implemented with high fidelity outside of the research context.

This study was conducted to explore the fidelity and feasibility of a classroom-based physical activity (ACTI-BREAK) program through process evaluation. Outcomes of the intervention have been reported separately [18].

## Methods

### Design

The ACTI-BREAK intervention was designed in consultation with current primary school teachers, and involved classroom teachers incorporating 3 × 5-minute active breaks into their classroom routine daily [19]. Additional detail on the intervention development is provided in the trial protocol [19]. The intervention aimed to improve on-task behaviour, academic achievement and school-based physical activity levels [19]. A 6-week pilot cluster randomised controlled trial assessed efficacy showing the intervention was effective for improving on-task behaviour but not academic achievement or physical activity levels. The current study utilised qualitative (interviews and focus groups) and quantitative data (questionnaire and accelerometry) to conduct a process evaluation. The trial is registered with Australia New Zealand Clinical Trials Registry (ACTRN12617000602325).

### Participants and setting

Children in Years 3 and 4 (*n* = 374; 74% response) and their teachers (*n* = 18) were recruited from six primary (elementary) schools across Melbourne, Australia for the pilot trial. Schools were randomised to either intervention (*n* = 3) or wait-list control (*n* = 3) group. The current study focuses on the responses of students (*n* = 138; 50% male; mean age 9.22 (SD = 0.61) years) and their teachers (*n* = 7; male: *n* = 2; female: *n* = 5) in the ACTI-BREAK intervention group.

### Measures

#### Fidelity of implementation

Teachers completed a log of the date and time they completed ACTI-BREAKS each day. As all ACTI-BREAKS were designed to be the same intensity level and to limit teacher burden, teachers were not required to log which activities they chose for their class. Children wore Actigraph GT3X accelerometers capturing physical activity data in 15-s epochs [20] at baseline, weeks 3 and week 6 of the intervention. Freedson’ cut-points were used to classify time spent in light- (> 25 to < 555 counts/15-s) moderate- (≥555 to <1034counts/15-s) and vigorous-intensity physical activity (> 1034 counts/15-s), as well as total physical activity (sum of all intensities) [21]. Accelerometer data were matched with teacher logs to assess intensity of physical activity during each ACTI-BREAK. As reported start and end times on teacher logs may not have been precise, and to ensure physical activity during the whole 5-min ACTI-BREAK was captured, 15 min of data (including 5-min before and after the reported ACTI-BREAK time) were extracted. Including the 5 min window either side of each ACTI-BREAK is unlikely to influence results as research has shown that the majority (65%) of the school day is spent sedentary, with only 5% spent in moderate- to vigorous-intensity physical activity [22]. Data were not extracted for reported ACTI-BREAK sessions adjacent to break times. Change in school day physical activity from baseline to mid- and baseline- to end-intervention was also explored to determine whether implementation was sustained over the intervention duration.

### Teacher evaluation of feasibility

#### Acute effects on behaviour

During week 3 of the intervention, the acute effect of active breaks on behaviour was recorded through teacher observations at the individual level (for students with parent consent) using a tool adapted from the Direct Behaviour Rating Scale [23] and at the whole class level (no identifying information was collected) using a modified version of the Classroom Behaviour and Assets Survey-Teacher Behaviour [24]. Group and individual behaviour were assessed simultaneously for a 10-min period immediately before and after participation in 3xACTI-BREAKS on 3 separate days.

#### Teacher interviews

In the week following intervention completion, teachers took part in a semi-structured telephone interview with a researcher (AW), designed to elicit discussion on factors affecting implementation of ACTI-BREAKS. Interviews lasted approximately 20–30 min and were audio-recorded and transcribed verbatim.

### Student evaluation of satisfaction

#### Enjoyment ratings

During each of weeks 1, 3 and 6 of the ACTI-BREAK program, teachers asked students to indicate their enjoyment of 3 different ACTI-BREAK sessions immediately following participation. Enjoyment was indicated on a 4-point Likert scale (I hated it = 1 to I loved it = 4) collected anonymously on paper forms, which students placed in a sealed box.

#### Satisfaction questionnaire

Upon completion of the ACTI-BREAK program, students completed a 13-item questionnaire to rate their satisfaction with the programme (refer to Table 2 for specific questionnaire items). Using a 4-point Likert scale (strongly disagree = 1 to strongly agree = 4) students indicated agreement with statements regarding (1) enjoyment of the ACTI-BREAKS (3 items): (2) preferred dose (frequency, intensity and duration) of active breaks (6 items): (3) effect on learning and behaviour (3 items): and (4) ability to do the activities (1 item). Students had the opportunity to provide additional feedback by answering the following open-ended questions: ‘*What did you like about the ACTI-BREAK program?’; ‘Was there anything you did not like about the ACTI-BREAK program?’*; and *‘Is there anything else you would like to say about the ACTI-BREAK program?’*

#### Focus groups

At intervention conclusion a random selection of students from each class were invited to participate in a semi-structured focus group discussion facilitated by a researcher (AW). Fifteen focus groups were conducted, each lasting approximately 30 min; 2 to 3 focus groups per class, with 4 to 6 students per group, and similar numbers of boys and girls in each. Focus groups were audio-recorded and transcribed by a commercial service. Questions were designed to provide a deeper exploration of participant satisfaction with the program. For example, to elicit discussion on factors effecting enjoyment of ACTI-BREAKS, students were asked to write down the names of two of their most favourite and two of their least favourite ACTI-BREAKS, and were then asked: *‘Can you tell me what you particularly liked/did not like about that one?’*

### Analysis

Quantitative data were analysed using Stata v15 (StataCorp, USA). Frequencies were reported. Change in physical activity from baseline- to mid- and baseline- to end-intervention and acute effects of ACTI-BREAKS on on-task classroom behaviour were analysed using paired *t*-tests.

Qualitative data were analysed using Braun and Clarkes 6 phases of thematic analysis [25] and NVivo 11 (QSR International Pty. Ltd.). This involved two researchers independently coding four transcripts (2 x student focus groups; 2 x teacher interviews). The minimal number of discrepancies were resolved through discussion until 100% agreement was reached. Remaining transcripts were coded by one researcher. Coded transcripts were repeatedly revised to ensure codes were consistently applied across transcripts. Similar codes were then grouped into higher order categories to create themes and subthemes. Themes were continually refined, and then defined and named. To ensure rigor of the data, all authors were involved in confirming final themes.

## Results

### Fidelity

Teacher logs indicated fidelity was fair in terms of required frequency, with an average of two ACTI-BREAKS completed per day and the number of days on which three ACTI-BREAKS were achieved ranging from 4 (*n* = 1 class) to 23 (*n* = 3 classes) out of 30 days (Table 1). Accelerometer data indicated fidelity was low in terms of children achieving prescribed moderate-intensity physical activity. The majority of ACTI-BREAK time was spent in light-intensity physical activity. Paired *t*-tests showed school day physical activity in the intervention group increased from baseline to mid-intervention (mean diff = 2.46;95% CI:0.64,4.29), but not baseline to end-intervention (mean diff = 1.93;95% CI:-0.20,4.05).

**Table 1:** Daily number and intensity of ACTI-BREAKS

	ACTI-BREAKS per day		LPA (mins/ACTI-BREAK)^a^		MPA (mins/ACTI-BREAK)^a^		VPA (mins/ACTI-BREAK)^a^		Total PA (mins/ACTI-BREAK)^a^	
	Mean	Range	Mean (SD)	Range	Mean (SD)	Range	Mean (SD)	Range	Mean (SD)	Range
Class 1	1.6	0 to 3	3.9 (2.3)	0.0 to 8.0	0.6 (0.6)	0.0 to 2.5	0.4 (0.5)	0.0 to 2.4	4.8 (3.1)	0.0 to 9.5
Class 2	1.7	0 to 3	5.8 (1.6)	1.6 to 8.6	0.8 (0.5)	0.0 to 2.2	0.2 (0.2)	0.0 to 1.0	6.8 (1.9)	1.6 to 10.6
Class 3	1.7	0 to 3	4.5 (2.2)	0.0 to 7.8	1.2 (0.8)	0.0 to 2.9	1.0 (0.8)	0.0 to 2.9	6.7 (3.3)	0.0 to 11.4
Class 4	1.4	0 to 3	5.8 (1.7)	2.0 to 11.0	1.2 (0.8)	0.0 to 4.0	0.7 (0.9)	0.0 to 4.5	7.7 (2.3)	2.0 to 13.4
Class 5	2.5	0 to 3	6.8 (1.7)	3.5 to 10.9	1.5 (0.8)	0.0 to 3.5	1.0 (0.7)	0.0 to 3.1	9.3 (2.2)	4.0 to 14.9
Class 6	2.5	0 to 3	5.2 (2.2)	0.3 to 10.5	1.6 (1.0)	0.0 to 4.5	1.3 (1.9)	0.0 to 9.3	8.1 (3.3)	0.3 to 14.8
Class 7	2.5	0 to 3	4.4 (2.0)	0.0 to 10.3	1.5 (1.3)	0.0 to 6.5	1.1 (1.2)	0.0 to 6.5	6.9 (3.3)	1.8 to 15.0
All classes	2.0	0 to 3	5.2 (2.2)	0.0 to 11.0	1.3 (1.0)	0.0 to 6.5	0.9 (1.2)	0.0 to 9.3	7.5 (3.1)	0.0 to 15.0

^a^ *15 min of data (including 5-min before and after the reported ACTI-BREAK time) were extracted*

*LPA* light-intensity physical activity, *MPA* moderate-intensity physical activity, *VPA* vigorous-inteniaty physical activity, *PA* physical activity, *SD* standard deviation

### Teacher evaluation of feasibility

#### Acute effects on on-task classroom behaviour

Teacher-reported on-task classroom behaviour at the individual level improved immediately following participation in ACTI-BREAKS (pre:74.78 vs. post:79.73; t = 4.75; *p* < 0.001). There was no change in behaviour at the class level (pre: 4.90 vs. post:5.50; *t* = 2.15; *p* = 0.07).

#### Interviews

Overall, teachers were positive in their post-intervention interviews and indicated they would continue to use the program. However, due to a number of barriers and facilitators identified, teachers indicated they would modify the program to suit their needs.

##### Barriers

Two major barriers to implementation were identified by teachers: (1) return to task; and (2) scheduling. Two teachers stated that students with behavioural challenges needed lower intensity active breaks to be able to settle back to work afterwards. All teachers described ACTI-BREAKS that provided clear directions to students and restricted movement as working best in terms of ease of settling the class after the ACTI-BREAK. Qualitative results confirmed that all teachers struggled to implement all three ACTI-BREAKS every day. When asked *“How did you find doing three ACTI-BREAKS every day?”* all teachers reacted along the lines of “*that was too many”* and commented that it would work better on an as needs basis. Scheduling around specialist classes was also often reported as a major barrier to implementation.

##### Facilitators

Three teachers shared that they tended to mostly repeat the ACTI-BREAKS that their classes enjoyed. Most teachers noted the task cards which explained how to do the ACTI-BREAKS made implementation quick and easy. The majority of teachers (6 out of 7) stated that having the flexibility to adapt the ACTI-BREAKS to suit their individual classes’ facilitated delivery. One teacher suggested that having the option to integrate active breaks into the curriculum (i.e. lesson content) would be helpful in terms of overcoming time constraints and pressure to get through the required curriculum. Another teacher reported that the size of the classroom and classroom furniture limited the available classroom space and suggested that having an option to do the activities outdoors would have worked better.

### Student satisfaction

#### Enjoyment of individual ACTI-BREAKS

As teachers selected the specific ACTI-BREAKS to be rated by students, not all ACTI-BREAKS were rated by all classes. Data were provided for 17 out of a potential 30 different ACTI-BREAKS. Enjoyment ratings indicated students liked or loved the majority (79%) of ACTI-BREAKS.

#### Satisfaction questionnaire

The majority of students agreed or strongly agreed that they enjoyed (96%), looked forward to (85%), and that their teacher enjoyed (78%) the ACTI-BREAKS (Table 2). About two thirds agreed or strongly agreed that it was easier to concentrate and that their school work improved after doing the ACTI-BREAKS. However about one quarter said they found it difficult to calm down after ACTI-BREAKS. Approximately half of all students thought they were too short and wanted more ACTI-BREAKS every day.

**Table 2:** Student satisfaction questionnaire results (*n* = 119–121)

Questionnaire item	Strongly disagree	Disagree	Agree	Strongly agree
	%			
Enjoyment				
*I enjoyed ACTI-BREAKS*	0.8	5.0	38.3	55.8
*I looked forward to ACTI-BREAKS*	2.5	12.6	32.8	52.1
*My teacher enjoyed ACTI-BREAKS*	4.2	18.3	52.5	25.0
Ability				
*I could do the ACTI-BREAK activities*	0.8	6.6	30.6	62.0
Dose				
*ACTI-BREAKS went for enough time*	15.1	23.5	36.1	25.2
*We had enough ACTI-BREAKS every day*	10.8	35.8	26.7	26.7
*ACTI-BREAKS didn’t go for long enough*	23.3	30.8	23.3	22.5
*We had too many ACTI-BREAKS every day*	48.7	35.3	9.2	6.7
*ACTI-BREAKS went for too long*	45.0	39.2	7.5	8.3
*We didn’t have enough ACTI-BREAKS every day*	22.5	25.8	33.3	15.3
Effect on learning and behaviour				
*I found it easier to concentrate after ACTI-BREAKS*	12.5	21.7	37.5	28.3
*My school work improved after ACTI-BREAKS*	8.3	25.0	42.5	24.2
*I found it hard to calm down after ACTI-BREAKS*	31.9	41.2	13.5	13.5

Open-ended results from the satisfaction questionnaire highlighted enjoyment of the program. Eight children did not complete these questions. Of those who did, there were a range of positives reported to the question *“What did you like about the ACTI-BREAK program”*, the most prevalent being that the activities were fun (*n* = 30), they helped them to learn better (*n* = 7) and that they got an opportunity to be active (*n* = 10). When asked *“Was there anything you did not like about the ACTI-BREAK program”* most students indicted there was not anything they did not like (*n* = 57). Other responses were generally positive, indicating students wanted the activities to go for longer (*n* = 6), and more often (*n* = 5). Some students reported that some of the activities were boring (*n* = 5), there was not enough space to move (*n* = 2) and that they would prefer to do the activities outside (*n* = 3). Other students disliked it when students did not calm down after doing ACTI-BREAKS (*n* = 9).

#### Focus groups

Participants identified a number of factors related to enjoyment, preferred dose of ACTI-BREAK and ability to return to task. Students particularly enjoyed those ACTI-BREAKS that incorporated choice (9 out of 15 focus groups) imagination (10 out of 15 focus groups) and challenge (9 out of 15 focus groups) and did not like ACTI-BREAKS that evoked silliness (8 out of 15 focus groups) or that they perceived to be too difficult (12 out of 15 focus groups), and some did not like doing ACTI-BREAKS in the confined space of their classroom (5 out of 15 focus groups). Some students reported wanting shorter duration ACTI-BREAKS so they had more time to spend on their school work (6 out of 15 focus groups). Students who reported playing sport wanted longer, more frequent ACTI-BREAKS at a higher physical activity intensity, often due to perceived fitness benefits (3 out of 15 focus groups). Students reported varying responses to ACTI-BREAKS in terms of ability to return to task, from feeling no difference to feeling calmer and more “switched on”. For some students (4 out of 15 focus groups) feeling tired after ACTI-BREAKS was identified as key to helping students do their work (3 out of 4 focus groups), while other students noted that feeling tired after hindered their ability to do their work (3 out of 4 focus groups).

## Discussion

This study is one of the first to report a process evaluation of a classroom-based active break intervention aimed at improving academic and physical activity-related outcomes. The intervention was shown to be feasible and generally a positive experience for teachers and students. However, some minor modifications in terms of the required frequency and intensity of ACTI-BREAKS could improve fidelity. Two major barriers to implementation identified by teachers were scheduling and ability for students to return to task. Facilitators to implementation were flexible delivery options, ease of implementation, and student enjoyment. Teacher reports of classroom behaviour showed on-task behaviour improved immediately following ACTI-BREAKS. Students were largely satisfied with the program, and particularly enjoyed ACTI-BREAKS that incorporated choice, imagination and challenge. Students did not enjoy ACTI-BREAKS that evoked silliness or were perceived as too difficult, and some did not like doing ACTI-BREAKS in the confined space of their classroom.

Data from teacher logs showed that fidelity was fair in terms of meeting the required frequency of ACTI-BREAKS. Scheduling was consistently identified as a barrier to achieving all three active breaks every day, and similar to previous studies was often associated with time constraints (e.g. fitting active breaks in around learning in other key curriculum areas) [13, 14, 17]. During the ACTI-BREAK development phase, teachers considered three short active breaks per day to be feasible. However, in practice teachers stated it was not always necessary to perform an active break (e.g. due to transition to specialist classes forming a natural break in the schedule, or students were working well). Consequently, consistent with findings from a previous study [14] teachers stated implementation would work better on an as needs basis. Thus, some flexibility around implementation (i.e. structured vs. incidental) and usage frequency may be necessary when developing future active break interventions.

Teachers suggested that the option to integrate ACTI-BREAKS into lesson content could help overcome time constraints associated with academic accountability. Such interventions have been shown to improve classroom behaviour [26, 27] and physical activity levels [28, 29] following participation, and can achieve the same physical activity intensity as active breaks [28–31]. However, it was thought that curriculum-focussed active breaks or physically active lessons would require teachers to change their teaching practices which could be met with resistance, and thus this was decided against. Additionally, due to known time constraints within busy teacher schedules, during the development phase it was decided for active breaks to be conducted inside the classroom to avoid taking children to another location which takes time [14]. However, some students and one teacher commented that having to perform activities within the confined space of the classroom was a limitation of the program. Thus, it may be necessary to provide teachers with a range of options for integrating physical activity into school day, including outdoor options, and the incorporation of academic content so that they can choose the option(s) that best suits their needs.

In addition to not meeting the prescribed frequency, teachers also generally did not achieve the prescribed moderate-intensity for the active break. This may be due to a failure of the intervention development as teachers could choose less intense options (e.g. ask children to creep around the room rather than gallop around). Thus, the program may need to be more prescriptive to ensure examples are all moderate intensity physical activity. Additionally, it may be important to provide further support for teachers so that they have the skills and confidence to manage classes during active breaks at a higher intensity, as physical activity of at least moderate-intensity is preferable to light-intensity physical activity in terms of health benefits [1].

An alternative explanation for implementation at a mainly light-intensity may relate to teacher concerns for moderate-intensity active breaks to have an adverse effect on behaviour. While in the development phase teachers considered moderate-intensity active breaks to be feasible [19], results of this study suggest that in practice teachers prefer light-intensity active breaks, perhaps due to the perception that came out in the interviews that students (particularly those with behavioural challenges) were easier to settle following light-, compared with moderate-intensity active breaks. In contrast, previous studies have consistently reported moderate- to vigorous-intensity active breaks had a positive acute effect on behaviour [6] and one indicated that behaviour improved most for those the most off task prior to active break sessions [27]. However, as that study [27] did not explore fidelity of implementation it is unclear whether the prescribed moderate-to vigorous-intensity physical activity was met – it is possible that intervention was also implemented at a mainly light-intensity. No other studies have considered the effect of light-intensity active breaks on behaviour, and only one has compared intervention effects based on behaviour prior to active break sessions [27]. Thus, future studies may consider comparing the effect of light- versus moderate-intensity active breaks on behaviour, as well as whether effects differ by behaviour prior to such sessions.

While teachers suggested the ability for students to return to task following ACTI-BREAKS was due to intensity of active break and whether or not students had behavioural challenges, students suggested that tiredness was key to settling back to work (or not) following ACTI-BREAKS. Some students reported that tiredness helped, while others reported that tiredness hindered their ability to return to task following ACTI-BREAKS. While in the current study active breaks were mostly implemented at a light-intensity, there was considerable variation in between students in the actual physical activity intensity achieved. The cognitive effects of acute bouts of physical activity have been shown to differ with physical activity intensity [32]. Specifically emerging research suggests light- to moderate- intensity physical activity benefits, while vigorous- intensity physical activity has no effect [32] or an adverse effect [33] on cognitive function immediately following sessions, perhaps due to exercise induced fatigue [34]. Thus, in the current study greater levels of tiredness may be associated with performing ACTI-BREAKS as a higher physical activity intensity and consequently an impaired ability to return to task, while lower levels of tiredness may be associated with performing ACTI-BREAKS as a lower physical activity intensity and increased ability to return to task. However, this assertion remains speculative.

In addition to ACTI-BREAKS that did not cause behaviour disruptions, similar to previous studies [35] teachers had an affinity to ACTI-BREAKS that students enjoyed. Students reported enjoying ACTI-BREAKS that incorporated choice, imagination and challenge, and disliking activities that were perceived as too difficult, evoked silliness, and some did not like performing ACTI-BREAKS in the confined space of their classroom. Additionally, the preferred dose of active break was different for different students. These findings are mostly new to the active break literature, with only one previous process evaluation exploring student perceptions of active breaks [17]. Similar to findings from a the current study, that study [17] also reported that some students wanted longer duration active breaks (10 to 20 min), while others wanted shorter active breaks (5 min). However, longer duration active breaks may not be feasible due to time constraints [13, 14, 17]. Thus, active breaks may need to be differentiated in other ways to cater to different student preferences. For example, the incorporation of outdoor activities, as well as different levels of movements so students can choose the movement that best suits their ability (e.g. including movements that all students can do, as well as more challenging movements for those students who desire extension).

### Strengths and limitations

A limitation of this study was that fidelity and classroom behaviour data were reported by the same teachers as those implementing the program, so there was potential for reporting bias. While the intensity of ACTI-BREAKS was collected objectively, there was potential for inaccuracy in teacher reported times that ACTI-BREAKS were conducted. This was overcome by analysing data with a 5-min window either side of each reported ACTI-BREAK time to ensure the entire ACTI-BREAK was captured. However, this method meant that movement superfluous to the ACTI-BREAK was also captured. A further limitation was that not all children were represented in the focus groups due to the high number of participating children. However, all children were represented in the other student evaluation measures and themes were mostly similar across focus groups, suggesting there was consistency of opinion across participating children. Another limitation was that teachers had the choice of which ACTI-BREAK activities to implement, so not all classes participated in the same activities. The current study had several strengths, including the use of data from both students and teachers to ensure a comprehensive assessment of feasibility and fidelity, and the objective assessment of physical activity intensity.

## Conclusions

Results from the current study indicate the intervention was feasible and generally a positive experience for teachers and students. However, it was implemented at a lower intensity and frequency than prescribed due to teachers’ perceptions of time constraints and the ability for students to return to task following higher intensity active breaks. Thus, the ACTI-BREAK intervention requires some modifications regarding the required intensity and frequency of the ACTI-BREAKS to improve fidelity. This information can be used to develop more feasible active breaks programs, or used to inform the integration of physical activity into the classroom setting more broadly.

## Authors’ information

AW is an experienced primary school teacher, and holds a Master of Public Health from the University of Adelaide.
